# Home and wild food procurement were associated with improved food security during the COVID-19 pandemic in two rural US states

**DOI:** 10.1038/s41598-024-52320-z

**Published:** 2024-02-01

**Authors:** Meredith T. Niles, Ashley C. McCarthy, Jonathan Malacarne, Sam Bliss, Emily H. Belarmino, Jennifer Laurent, Scott C. Merrill, Sarah A. Nowak, Rachel E. Schattman

**Affiliations:** 1 Department of Nutrition and Food Sciences, University of Vermont, 355 Carrigan Wing, 109 Carrigan Drive, Burlington, VT 05405 USA; 2 Gund Institute for Environment, University of Vermont, Burlington, VT USA; 3 School of Economics, University of Maine, Orono, ME USA; 4 Rubenstein School of Natural Resources, University of Vermont, Burlington, VT USA; 5 Department of Nursing, University of Vermont, Burlington, VT USA; 6 Department of Plant and Soil Sciences, University of Vermont, Burlington, VT USA; 7 Department of Pathology & Laboratory Medicine, University of Vermont, Burlington, VT USA; 8 School of Food and Agriculture, University of Maine, Orono, ME USA

**Keywords:** Nutrition, Public health, Quality of life, Psychology and behaviour, Socioeconomic scenarios

## Abstract

Both food insecurity and home and wild food procurement (HWFP), including gardening, increased in many countries during the COVID-19 pandemic; yet little evidence has demonstrated what impact HWFP had on food security. Using data from a representative sample of nearly 1000 residents in the two most rural US states (Vermont and Maine) conducted via an online survey in Spring/Summer 2021, as well as matching techniques, we compare food security outcomes among households who did and did not participate in HWFP in the first year of the pandemic. Nearly 60% of respondents engaged in HWFP in some way during the first year of the pandemic, with food insecure households more likely to do HWFP. Furthermore, HWFP early in the COVID-19 pandemic is associated with improved food security in the 9–12 months later, though these improvements were primarily associated with newly, not chronically, food insecure households. Newly and chronically food insecure households were more likely to want to continue these activities in the future, but also exhibited greater barriers to land access and costs associated with these activities. These results suggest that HWFP may provide food security improvements for certain households that utilize them, especially during crisis situations. Future research about HWFP should continue to explore multiple HWFP strategies, their barriers, and their potentially myriad relationships to food security, diet, and health outcomes, especially with longitudinal data.

## Introduction

Producing or obtaining one’s own food via gardening, fishing, foraging, hunting, raising animals, and/or preserving food (hereafter called home and wild food procurement (HWFP)) may have important effects on food security and dietary intake. Most prior research has focused on gardening (both home and community), which has been shown to increase food security^1–3^, increase fruit and vegetable consumption^1,4^, reduce food costs^5^, and provide additional income-generating opportunities^3^. However, the prevalence of other HWFP activities (e.g. fishing, hunting, foraging) and their impact on food security is less understood, especially outside of indigenous communities^6^. Furthermore, much of the existing evidence consists of small-scale studies (e.g.,^7–9^) and qualitative case studies^10^, with calls for more quantitative population-level studies^11^. As a result, population-level conclusions about the prevalence of HWFP and its implications for food security remain limited.

The onset of the COVID-19 pandemic brought with it a documented increase in HWFP and a new opportunity to explore its impact at scale. Several COVID-19-era studies have examined individuals’ increased interest in HWFP across diverse socioeconomic and political regions, including Canada^12,13^, Palestine^14^, Sri Lanka^15^ and Chile^16^. In the context of population-level disruptions to work, personal, and social lives, this literature finds various motivations for the growth in HWFP during the pandemic, including food security and supply chain concerns^17–19^, a desire to spend time in nature^13,14,20^, more free time^13^, seeking spaces of refuge and community (in community gardens)^20,21^, stress reduction or mental wellbeing^16,20^, and a perception that HWFP activities done in the outdoors were COVID-safe^22^. While most pandemic-related HWFP studies have focused on gardening, one study also documented an increase in urban foraging^23^. Additionally, previous work demonstrated increased participation, both in terms of rates and intensity, in fishing, foraging, hunting, raising backyard animals and canning during the first six months of the pandemic^6^.

While research since the onset of the COVID-19 pandemic has shown an increase in HWFP across disparate global regions, there remains very little evidence about the effects of this increase in HWFP on individuals and communities. The most common documented impacts of HWFP engagement during the COVID-19 pandemic include benefits to mental health^16,24^ and higher fruit and vegetable intake among those engaging in HWFP as compared to those who do not engage in HWFP^6,15^, though these associations have been measured only at single time points. Similarly, little is known about the extent to which HWFP activities have continued beyond the early days of the pandemic, when stay at home orders and quarantine were the norm, and many people had additional free time^13^.

Barriers to undertaking and sustaining HWFP are well documented, both before and during the pandemic. Previously studied barriers include inadequate land access^7,16^, limited knowledge of how to engage in HWFP practices^7,16,25^, shortages of supplies such as seeds^20^, and high levels of pests in gardening^8^. Such barriers are often enough to reduce, stop, or prevent HWFP altogether^26^. For example, Chenarides et al. (2021) identified a reduction in HWFP between 2017 and the beginning of the pandemic in 2020 in two urban gardens in Phoenix and Detroit US, suggesting the fragility of participation in such activities^27^.

At the outset of the pandemic, our research group deployed multiple rounds of surveys in two rural US states (Vermont and Maine) to assess how COVID-19 affected food security status, mental health, dietary intake, and other measures of wellbeing. This study builds on our previous work^6,24^ and expands our analysis to include data on HWFP engagement and food security from before the COVID-19 pandemic through the first year after its onset. Quantitative survey data collected from a representative population-level survey of nearly 1,000 respondents in Vermont and Maine was used to conduct our analysis using a series of statistical tests and matching techniques. We assessed the following research questions:

1. How did food security and HWFP prevalence change during the first year of the pandemic as compared to before the pandemic?
2. Did HWFP engagement during the first year of the pandemic correlate with improved food security outcomes, especially for households that were food insecure during the early part of the COVID-19 pandemic?
3. How likely are respondents to continue HWFP in the future? Who is most likely to intend to continue? What barriers do people face for HWFP and how does this differ by food security status?

## Results

### Demographic characteristics

A total of 988 individuals—426 in Vermont and 562 in Maine—responded to the survey. Survey respondents were representative of the Vermont and Maine populations in their race/ethnicity and education. There were no major differences in outcomes by state; thus, Vermont and Maine respondents were combined for this analysis. Table 1 details the demographic characteristics of the respondents.

**Table 1:** Demographic characteristics of survey respondents and population overall of Maine and Vermont from US Census data.

Demographic Characteristic	Sample (%)	n = 988	General Population (%)
Female	68.3	675	50.9
Male	30.3	299	49.1
Other gender identity/prefer not to respond	1.4	14	–
Non-Hispanic White	91.6	905	93.1
BIPOC	8.4	83	6.9
Income < $50,000	53.3	527	42.5
Income > $50,000	46.7	461	57.5
Job loss during pandemic	14.6	144	–
Bachelor's degree or higher	33.9	335	33.8
Rural	55.4	547	53.5
Urban	44.6	441	46.5

### Food security prevalence

**Figure 1:**
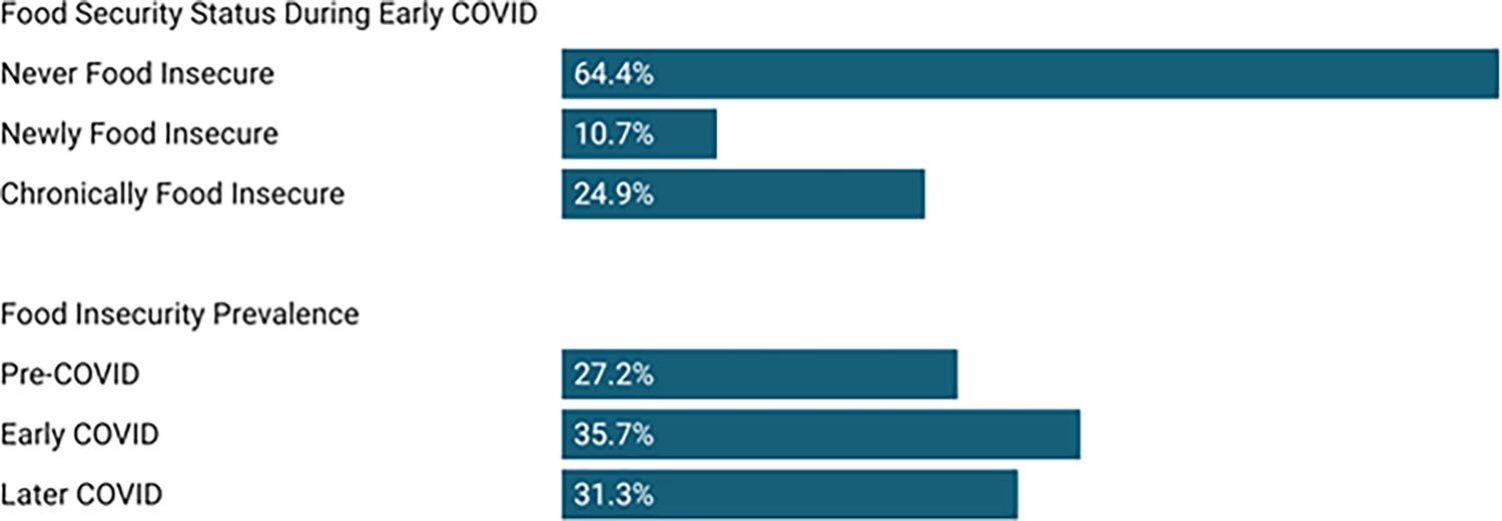
Percent of respondents classified by food security status during early COVID, and prevalence of food insecurity over time.

According to their retrospective responses to the USDA 6-item food security survey module a year into the pandemic, 27.2% of respondents’ households were food insecure pre-COVID-19 (i.e., during the year prior to March 2020), with 35.7% food insecure in early COVID (from March 2020 until the survey was taken in Spring/Summer 2021), and 31.4% food insecure in later COVID (the four months before taking the survey; see Fig. 1). In the first year-plus of the pandemic, 24.9% of respondents’ households experienced chronic food security (i.e., they were food insecure pre- and early COVID), while 10.7% were newly food insecure.

### Home and wild food procurement

**Figure 2:**
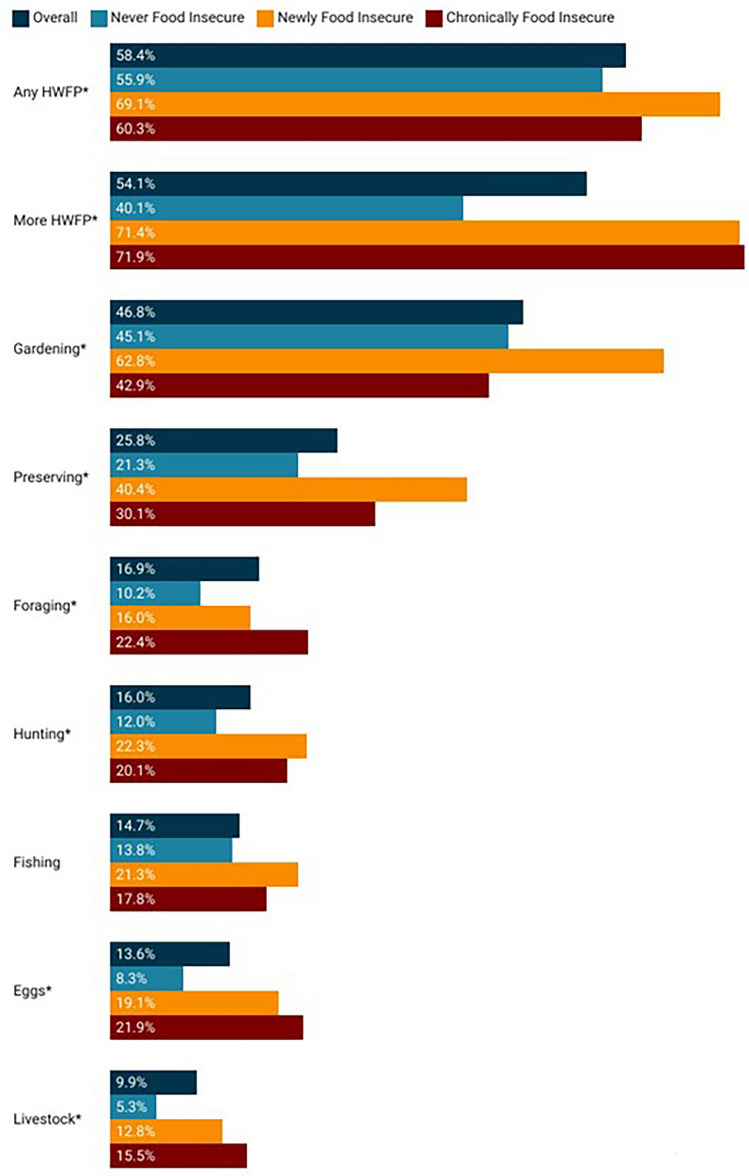
Home and wild food production during the first year of the COVID-19 pandemic and for specific activities based on food security status. *Indicates statistically significant difference (*p* < 0.05) across the three types of food security.

Nearly 60% of respondents indicated that they had engaged in some type of HWFP activity since March 2020, with 54.1% of those engaging in HWFP indicating that they either did so for the first time or did so more intensely since the start of the pandemic (Fig. 2). Gardening was the most frequently reported HWFP (46.8% of respondents), while the least frequently reported was raising livestock for meat or dairy (9.9%).

We found statistically significant differences in overall HWFP engagement since the onset of the COVID-19 pandemic, as well as in specific types of HWFP engagement, across food security status. Overall, food insecure households were significantly more likely to engage in HWFP since the beginning of the pandemic as compared with food secure households. Newly food insecure respondents were the most likely (69.1%) to engage in HWFP. Newly and chronically food insecure households were also significantly more likely than food secure households to engage in HWFP for the first time or more intensely since the beginning of the pandemic (*p* < 0.001). Among specific activities, food insecure households were significantly more likely than food secure households to engage in all individual HWFP activities with the exception of fishing (*p* = 0.101). Newly food insecure households were significantly more likely to garden since the beginning of the pandemic (62.8%) but food secure and chronically food insecure households gardened at nearly equivalent prevalences (45.1% and 42.9% respectively).

### Changes in food insecurity associated with HWFP

As expected, based on the distribution of food insecure households engaging in different HWFP activities, our first matching analysis (Supplementary Table 2; robustness checks in Supplementary Table 6) identified positive associations between overall engagement in HWFP and food insecurity during the first year of the pandemic (b = 0.077, *p* = 0.010). We found that specific HWFP activities (foraging (b = 0.172, *p* < 0.001), hunting (b = 0.014,  *p*= 0.003), livestock (b = 0.189, *p* = 0.003), eggs (b = 0.192, *p* < 0.001), and preserving (b = 0.127, *p * = 0.039)), were also positively associated with food insecurity since the onset of the COVID-19 pandemic. Furthermore, engaging in each specific HWFP activity more intensely or for the first time were all positively associated with food insecurity, as was engaging in *any* HWFP more intensely or for the first time (b = 0.176–0.405, *p* < 0.05) (Supplementary Table 2). Looking only at later COVID food insecurity, neither gardening (b = 0.023, *p* = 0.441) nor engaging in any HWFP since the COVID-19 pandemic (b = 0.040, *p* = 0.179) were associated with food insecurity, but nearly all other activities—as well as engaging in them for the first time or more intensely—continue to be positively associated with food insecurity (b = 0.110–0.414, *p* < 0.05) (Supplementary Table 3; robustness checks in Supplementary Table 7).

We found more nuanced results exploring these relationships conditional on initial food security status (Supplementary Tables 4 and 5; robustness checks in Supplementary Tables 8 and 9). Among households that were food secure pre-COVID, those that engaged in HWFP during the pandemic were more likely to be food insecure in early COVID compared to those that did not. These results were also consistent for gardening, preserving food, and those engaged in each specific HWFP activity more intensely or for the first time (Supplementary Table 4).

Among households that were food insecure pre-COVID, however, those that engaged in HWFP during the pandemic were not more likely to be food insecure than those that did not engage in HWFP. Taking the analysis one step further, we examined whether early COVID food insecure households who engaged in HWFP at that time changed their food security status in later COVID. Among households that experienced food insecurity early in the pandemic, those who engaged in HWFP were more likely to be food secure later in the pandemic compared to those who did not engage in HWFP (b = − 0.079, *p* = 0.040) (Supplementary Table 5). We found the same result for households that were food insecure in early COVID and gardened (b = − 0.110, *p* = 0.008) or foraged more than before or for the first time (b = − 0.133, b = 0.016): they were more likely to be food secure in later COVID than those who did not. Thus, HWFP engagement in early COVID was associated with improved food security outcomes for food insecure households in the 9–12 months after the onset of the pandemic.

**Figure 3:**
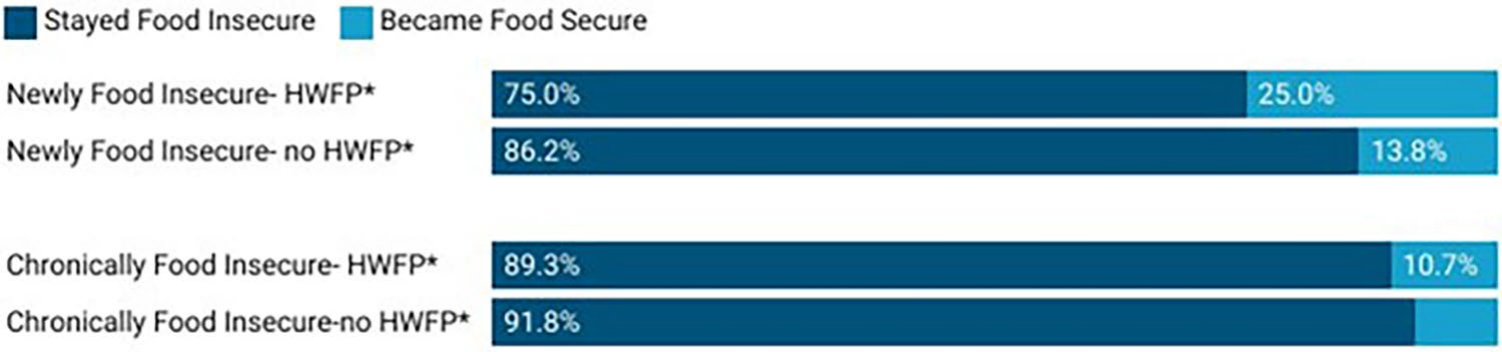
Change in food security status (in the four months prior to the survey) among newly and chronically food insecure households engaging or not in HWFP. *Indicates statistically significant difference (*p* < 0.05) between food security improvement by HWFP status.

These improvements in food security were primarily associated with newly food insecure households. Among newly food insecure households, 21.5% became food secure in later COVID, while only 9.7% chronically food insecure households became food secure (p = 0.005). Furthermore, when examining these changes by HWFP participation (Fig. 3), we found that newly food insecure households that engaged in HWFP had the highest conversion to food security in later COVID (25.0%), as compared to chronically food insecure households also engaging in HWFP (10.7%,), newly food insecure not doing HWFP (13.8%) and chronically food insecure not engaging in HWFP (8.2%) (p = 0.010).

### Likelihood to continue HWFP and barriers to HWFP

**Figure 4:**
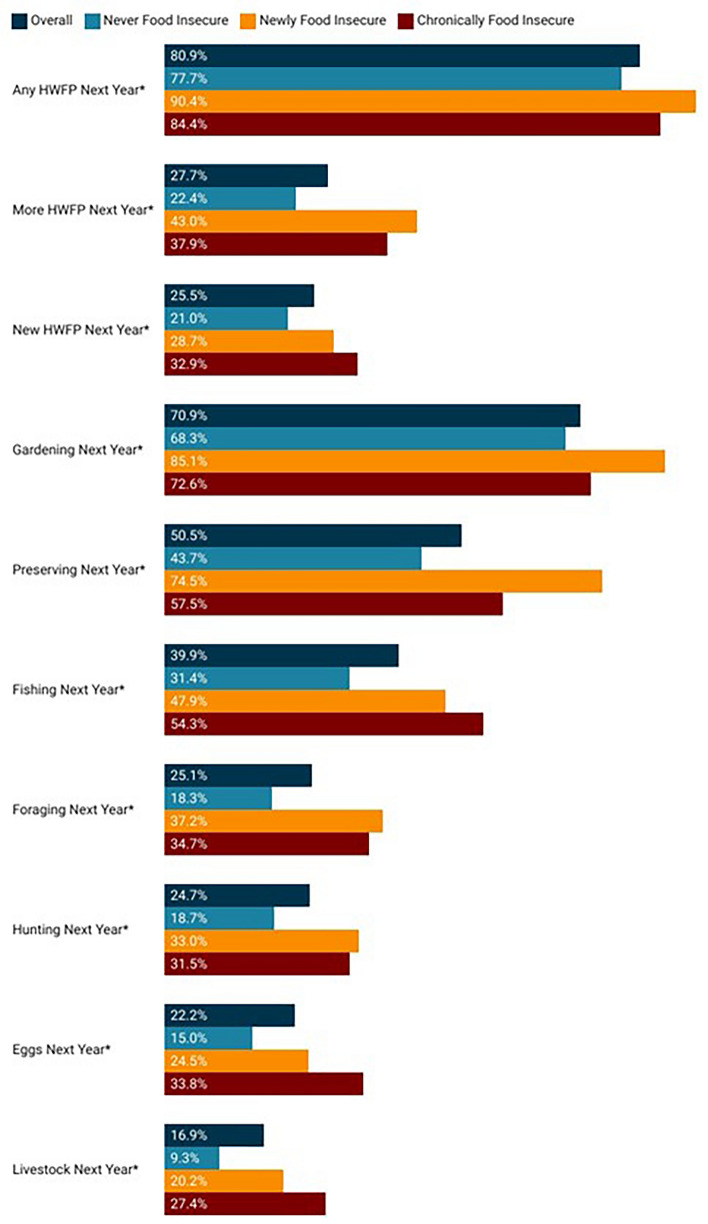
Respondent reports of intent to continue HWFP activities and intensity in the 2021–2022 year. *Indicates a statistically significant difference (*p* < 0.001) among outcomes by food security status.

We also examined the likelihood of respondents to continue HWFP in the coming year (2021–2022). Overall, 80.7% of respondents indicated they intended to engage in some type of HWFP activity in the coming year, with engagement in gardening being the most likely (70.9%) (Fig. 4). Just over one-quarter of respondents indicated they would try a new HWFP activity in the upcoming year (27.7%) or engage in at least one of their existing HWFP activities more than previously (25.5%). Overall, both newly and chronically food insecure households were significantly (*p* < 0.001) more likely to intend to continue all types of HWFP in the coming year, and to increase their engagement in those activities.

**Figure 5:**
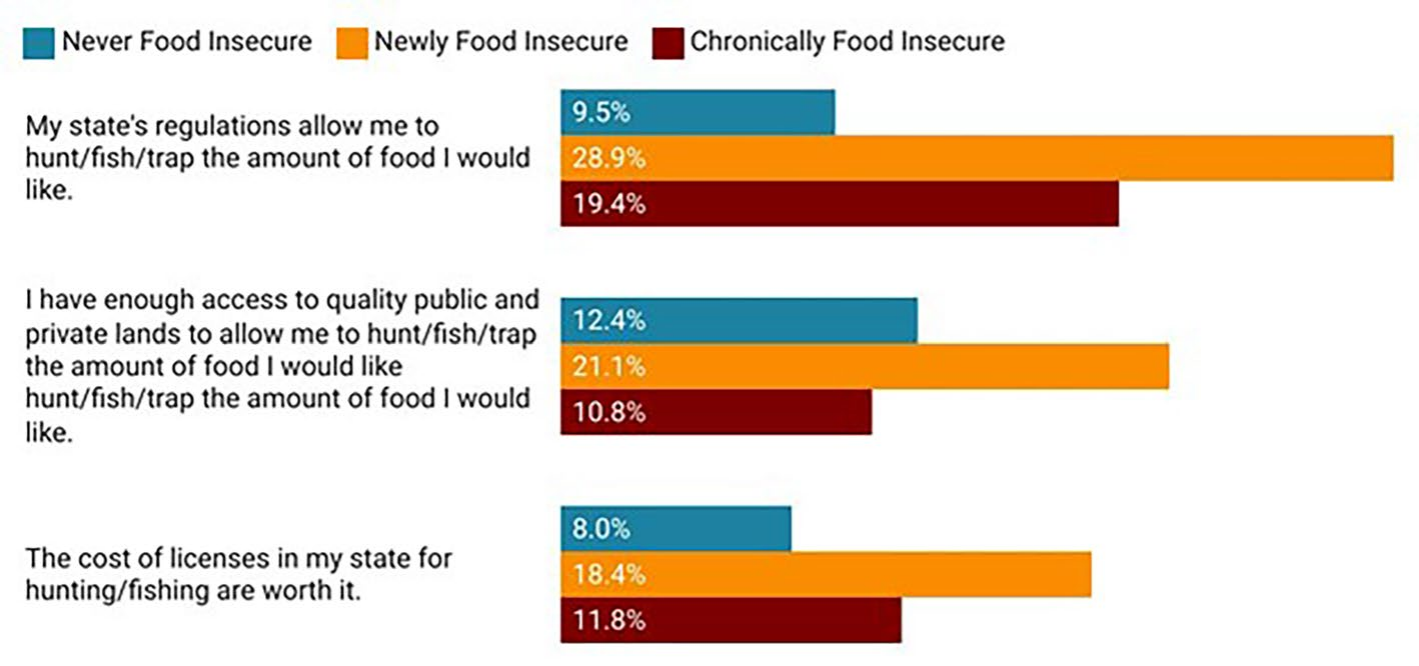
Disagreement with statements about potential barriers to HWFP by food security status, among those engaging in HWFP. Percentages indicate the percent “somewhat” or “strongly” disagreeing with the statement.

Finally, we asked a few questions related to potential barriers that people face engaging in HWFP, including about restrictions related to regulations within the state, land access, and the cost of licenses for hunting and fishing (Fig. 5). Overall, we find that chronically and newly food insecure households engaging in HWFP are more likely to disagree that they have enough access to quality public and private lands for obtaining food for hunting, fishing, and trapping (never food insecure = 12.4%, chronically food insecure = 10.8%, newly food insecure = 21.1%, *p* = 0.042), and that the cost of licenses in the state are worth it (never food insecure = 8.0%, chronically food insecure = 11.8%, newly food insecure = 18.4%, *p* = 0.020), as compared to respondents that were never food insecure.

## Discussion

Despite the growth in research exploring HWFP during the COVID-19 pandemic, this work is among the first to directly correlate HWFP during the pandemic with improved food security outcomes for food insecure households. Our research also demonstrates that, among this sample of respondents in two rural New England states, most live in households that engaged in HWFP and engagement in these activities continued to grow during the first year of the pandemic, especially among food insecure households. This is particularly evident in comparison to previous results, which found that 35% of respondents, from a sample in the same region with similar demographic characteristics, engaged in HWFP during the first six months of the COVID-19 pandemic^6^. By comparison, 58% of respondents in this study engaged in some HWFP activity within the first year of the pandemic.

These comparisons also point toward important future research needs related to measuring HWFP at multiple time points throughout the year. For example, in our previous analysis, only 6.2% of respondents reported engaging in hunting from March to August 2020^6^; but the current study shows 16.0% of respondents' households engaged in hunting within the first year of the pandemic. The difference may be attributed to the fact that in Vermont and Maine, hunting seasons for most game species occur in the late fall and early winter, a timepoint which the first iteration of our survey failed to capture. Similar differences were observed for foraging, where only 9.2% of individuals participated in the first six months of the pandemic^6^, but 16.9% participated within the first year of the pandemic (accounting for fall foraging). Thus, as additional research on HWFP continues, especially that which focuses on multiple HWFP strategies, it is critical to consider the seasonality of those activities and design data collection that captures these factors.

These results add nuance to the existing research on HWFP and food security by separating out households that have been chronically food insecure from those that were newly food insecure. Evidence from the first year of the pandemic demonstrates that newly versus chronically food insecure households had different demographic and social experiences with food insecurity, which may have influenced their engagement in HWFP. A review of food security dynamics in the U.S.A. shows that chronic food insecurity is most likely to be experienced by non-white, less-educated individuals in households headed by women^28^. It is noted that it is difficult for current measures of food insecurity to fully capture the processes that lead to chronic food insecurity because survey modules ask about households’ experiences within the last 30 days or the last year. As such, there may be an opportunity to reconsider food security screening questionnaires to inquire about the length of time overall that households may have experienced food insecurity. At the same time, it is important to better understand the dynamic ways in which social and environmental shocks, like those experienced during the COVID-19 pandemic, influence the severity and persistence of food insecurity^27^.

Likewise, motivation to engage in HWFP can vary based on socio-demographic factors, prior experience, available time, and other factors, which not only affect whether an individual engages in HWFP, but how they do so. For example, a recent study found that women gardeners were more likely than men to plant a diversity of plant species, and that region of origin influences crop composition choices^29^. Whether someone engages in HWFP activities, such as hunting and fishing, as supplementary versus subsistence food acquisition behaviors can be influenced by cultural traditions, such as those held by some members of Tribal communities^29^. Furthermore, a study of countries in the European Union explored how HWFP varied between being largely recreational to being a coping strategy for food insecurity, with differential impacts on well-being^30^.

Our previous work identified that food insecure households were not more likely to engage in HWFP overall, but instead were more likely to be engaging in HWFP more intensely or for the first time since the onset of the pandemic^6^. However, this analysis found that the story is more complicated. Newly food insecure households are the most likely to have engaged in HWFP. Although, overall, chronically and newly food insecure individuals equally engaged in HWFP more intensely or for the first time, they engaged in certain, specific HWFP activities at significantly different levels. Newly food insecure individuals were significantly more likely to garden and preserve food for example, while chronically food insecure households were more likely to forage. The relative influence of gardening on improving food security outcomes, especially for the newly food insecure, may be a function of the amount of time, resources, or knowledge dedicated to the activity. Furthermore, our findings that newly food insecure households were best able to become food secure, especially those that gardened, also highlights that newly food insecure households may have had different levels of resources than chronically food insecure households, especially if they were not facing years of food insecurity.

Finally, we found that chronically and newly food insecure households engaging in HWFP were more likely to indicate they didn’t have enough access to quality public or private lands for engaging in HWFP and felt that the cost of licenses within their state were not worth it, as compared to never food insecure respondents. These findings echo previous evidence about inadequate land access and the costs of doing HWFP affecting engagement in these activities^7^. However, we acknowledge that there are many other potential barriers to HWFP^7^, and future research in this area should explore the barriers to HWFP by food security status, to better understand the ways to support HWFP activities by diverse households.

That newly food insecure households, especially those who gardened, were more likely to transition back to food security in the first year of the pandemic continues to add evidence to the existing body of research demonstrating that gardening correlates with improved food and nutrition security^7,31–33^. Our work strengthens these findings, particularly given our large sample size across representative demographics in two states, which builds upon the largely qualitative or non-representative structure of previous studies. Our work also demonstrates that improved food security is associated with HWFP and gardening both within crises as well as in “normal” times, which others have demonstrated (e.g.^1,3^).

The prevalence of HWFP during a crisis period is noteworthy, and important for future research. The global COVID-19 pandemic deeply affected the global economy and food systems^33,34^. As the direct financial and public health restrictions and impacts from the pandemic have waned, it remains unclear the extent to which the interest in HWFP will continue, especially as individuals resume employment and additional activities. The pandemic provided many people the opportunity to engage in new activities with time commitments that may not be feasible or desirable in non-pandemic crises^13^. Indeed, the relationship between HWFP and household food security (as measured by the USDA survey module) likely changed as “normal” life resumed and people regained assured access to income and groceries. At the same time, there are continued long-term impacts of the pandemic, including elevated levels of anxiety and depression^34,35^, which may motivate many people to continue HWFP for other mental health and wellbeing associated reasons^14,16,20^.

Future research may, and should, continue to track HWFP engagement as well as its contribution to food and nutrition security in the recovery from the pandemic. At the same time, additional quantitative research could assess how the food outputs from HWFP relate to dietary intake and related health outcomes. Most existing studies in the Global North have not examined how the percent of food obtained from HWFP influences food security and diet outcomes. Additional research in this vein could also more completely assess the varying potential impacts of HWFP engagement beyond food security. Indeed, other studies have demonstrated that HWFP is associated with improved mental health^24^, physical activity^36,37^, and social connectedness^38,39^; but rarely are the suite of these potential impacts explored together.

Ultimately, our work is limited in its ability to demonstrate causality because it is cross-sectional. The use of the USDA household food security survey module presents additional limitations: it relies on respondent recall, in this case more than one year into the past, and it essentially measures whether households had enough money for food during the recall period, which makes it an awkward tool for assessing the effects of food acquisition methods that do not involve paying money for food, such as HWFP. However, future studies using cohort models could understand how people engage in HWFP and its link to food security and other health and diet outcomes more concretely by tracking the same people and households over time. Furthermore, while our sample was representative on certain demographic characteristics, it was overrepresented by households making less than $50,000 annually (Table 1), which likely explains the higher level of food insecurity observed before and since the COVID-19 pandemic, which are corroborated by other similar studies^40^, but are higher than other national studies^41^. Longitudinal studies would also be able to better track changes in food security status within income groups over time.

## Conclusion

In this study, we showed how HWFP was used in different ways and intensities before and during the COVID-19 pandemic across two predominantly rural U.S. states. Our results reveal notable differences between segments that became food insecure during this period of upheaval and those that were chronically food insecure, such that newly food insecure households were better able to become food secure, especially those engaging in HWFP. This suggests that chronically food insecure households face greater barriers to both engaging in HWFP and improving their food security through these activities. Greater understanding of the barriers to HWFP faced by chronically food insecure households is critical for future work to enable potential improvements using these strategies. It is important to note that engagement in HWFP is dynamic, and changes that occurred during the pandemic may reverse toward a previous “normal”, solidify as a new stable state, or become exacerbated by continued political, socio-ecological or economic issues such as inflation, recession, or a resurgence of disease. Given the emerging evidence that HWFP can contribute to positive health behaviors and outcomes, our findings and future research have public health importance, with relevance to audiences interested in human health and wellbeing, as well as the social and environmental consequences of mainstream food systems. As such, we suggest that future research continue to explore HWFP over time following the pandemic to assess whether such strategies show long-term relationships to food security and other nutrition and health outcomes.

## Methods

### Data collection

Data collection was conducted in Spring/Summer 2021 in Vermont and Maine, USA, via an online cross-sectional survey. The survey builds on work by the National Food Access and COVID research Team (NFACT)^41^ and expands the set of questions related to HWFP participation and its barriers. The original NFACT survey underwent validation in Vermont with 25 respondents aged 18 and over^42^. The survey consisted of seven sections describing food sources; HWFP; food security, employment and COVID-19 experiences; dietary intake; health; physical activity; and demographic information. Institutional Review Board approval was obtained from the University of Vermont (IRB protocol 000000873) before beginning data collection and all methods were implemented in accordance with these IRB guidelines and regulations. Data was collected via Qualtrics (Provo, UT) research panels. We used recruitment quotas for our general population sample to ensure that the sample was representative of the populations of Vermont and Maine with respect to race and ethnicity, based on the most recent population profiles from the American Community Survey^43^. Informed consent was obtained from all participants prior to beginning the survey and data collection, and respondents could only participate once. Respondents were anonymous in the data collection process.

### Variables of analysis

We used four categories of variables in our analysis (Supplementary Table 1). These were: food security, home and wild food procurement since the COVID-19 pandemic (HWFP COVID), increased HWFP since the COVID-19 pandemic (HWFP More), and demographic characteristics.

**Figure 6:**
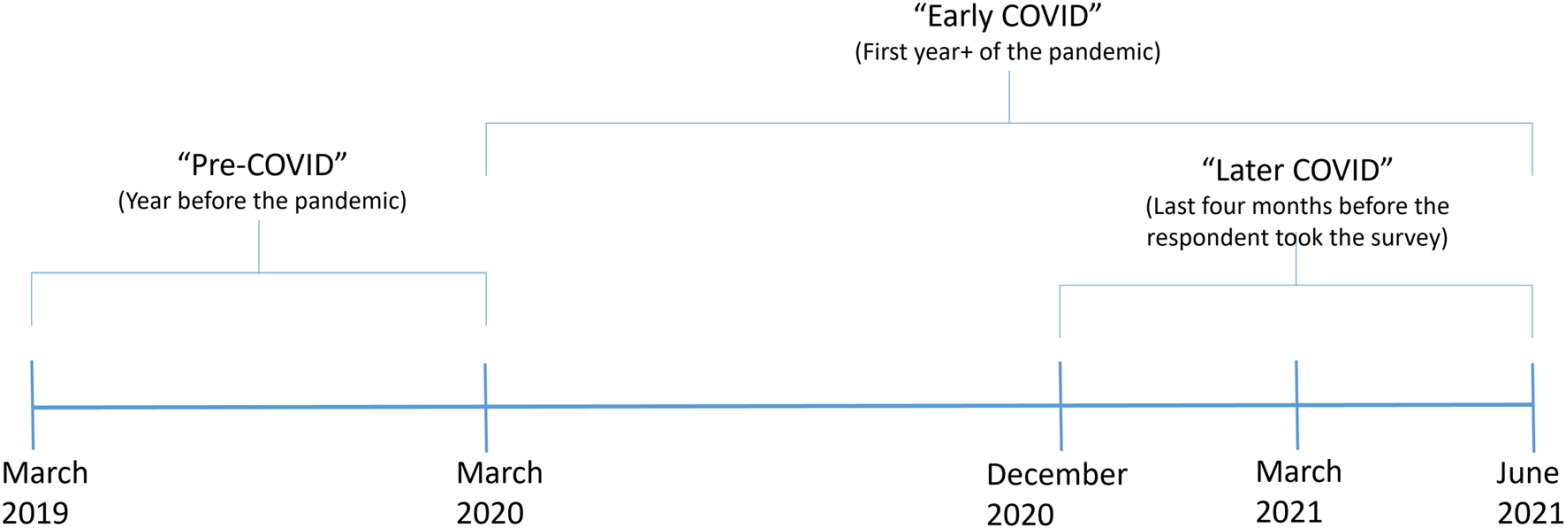
Time periods referenced for food insecurity in this article.

Food security status was measured using the six item short-form USDA household food security survey module^44^. Following the standard protocol for calculating food insecurity, respondents who responded affirmatively to two or more questions out of six were classified as food insecure. This binary food security measure was calculated during each of three time periods: (1) pre-COVID-19 pandemic (“Pre-COVID”- i.e., in the year before the COVID-19 pandemic); (2) early COVID-19 pandemic (“Early COVID”- i.e., in the first year of the pandemic); and (3) later COVID-19 pandemic (“Later COVID”- i.e., in the last four months before the survey, corresponding to Winter/Spring 2021) (Fig. 6). All food security questions were asked in the same survey, such that all questions were retrospective (as is typical within the USDA food security module), which may have resulted in errors due to recall bias. In addition, we generated a categorical variable with three categories of food insecurity: (1) never food insecure (before or during the COVID-19 pandemic), (2) newly food insecure (food secure before the pandemic, but food insecure in early COVID), and (3) chronically food insecure (food insecure both before and in early COVID).

In addition to these key variables, we utilized several variables related to HWFP, including “any HWFP since March 2020” and “increased HWFP since March 2020” (engaging in a HWFP activity for the first time or more than before). We also explored engagement in specific HWFP activities including gardening, fishing, foraging, hunting, raising livestock for meat or dairy, raising poultry for eggs, and preserving food. We inadvertently left “hunting more” out of our survey; therefore, the analyses where we explored engaging in HWFP more (for the first time or more intensely than before) include all activities except hunting. Finally, we include three questions related to regulatory, land access and cost barriers for engaging in HWFP.

We also report demographic characteristics of our respondents, including gender identity, race, ethnicity, income, job loss experienced during the pandemic, education, and rural/urban classification (as assessed using rural–urban commuting area (RUCA) codes)^44,45^.

### Statistical analysis and matching techniques

We employed several different statistical approaches to answer our research questions, with analyses conducted in Stata 17.0. We assume all missing data was missing at random, and we did not impute data for any analysis. Chi-Square tests were used to determine whether food insecure households were more likely to engage in HWFP and barriers to HWFP, and analysis of variance (ANOVA) to assess whether HWFP varied by food security status. We also used Kruskal Wallis tests (one-way ANOVA on ranks) to study future intention to engage in HWFP.

To further explore research questions 1 and 2, we employed a quasi-experimental matching method to assess whether HWFP engagement correlated with food security outcomes overall and whether HWFP resulted in improved food security outcomes from one time period to the next. Matching analysis is a statistical approach to conditioning on observables in order to identify the effect of a “treatment” that some individuals have received and others have not^46^. In our case, the treatment we are interested in was HWFP engagement. We employed matching for two analyses. First, we assessed whether HWFP engagement correlated with food security during early and later COVID periods. Second, we analyzed whether households that engaged in HWFP during early COVID had improved food security status in later COVID. Our analysis matched respondents engaging in HWFP to observably similar households not engaging in HWFP, with an aim of balancing the distribution of both observable and unobservable covariates in each group^47^. We matched on a set of respondent characteristics including: race/ethnicity (BIPOC or non-Hispanic White), income (households making less than or more than $50,000 annually), gender identity (male or female), job loss during the pandemic, bachelor’s degree, and rural/urban status. Our question related to gender identity included non-binary options; however, only a small sample indicated non-binary gender. Therefore, we utilize only male and female gender identities for our matching analysis.

We used a *k*- nearest neighbor matching approach, which uses the *k* most similar non-treated observations to create a comparison value for each treated observation. Previous research has demonstrated that this matching approach works well with eight or fewer covariates^46,47^. We report the total number of control, treated, and matched individuals in all our models to satisfy the common support condition^47^. Our primary results use the Mahalanobis distance between treated and non-treated observations to identify matches and weight comparison values. Mahalanobis distance works well with fewer covariates^46,48^, as well as in situations where such covariates may be correlated even if they use different scales^49^, as is the case with some of our covariates such as income and education. Given the discrete nature of our matching variables, most of our matches are exact. In order to assess the robustness of our primary results, we repeated our estimation, varying the minimum required matches (from five down to one) and requiring that all matches be exact matches (Supplementary Tables 7–9).

### Ethics approval and consent to participate

Ethical approval was obtained through the Institutional Review Board at the Universities of Maine and Vermont, and written consent was obtained from all participants.

### Supplementary Information

Supplementary Tables.

## Data Availability

The survey instruments for this work are publicly available at Harvard Dataverse (https://dataverse.harvard.edu/dataset.xhtml?persistentId=doi:10.7910/DVN/BIHEYJ). Individuals interested in data access should contact the corresponding author for further details.
