# Supplementary material for: Home and wild food procurement were associated with improved food security during the COVID-19 pandemic in two rural US states
Source: Sci Rep. 2024 Feb 1;14:2682. doi: 10.1038/s41598-024-52320-z (PMC10834469; doi:10.1038/s41598-024-52320-z)
Supplement: Supplementary file 1 — Supplementary Tables. [file 41598_2024_52320_MOESM1_ESM.docx]

**Home and wild food procurement associated with improved food security during the COVID-19 pandemic**

Supplementary Table 1. Variable Names, Questions and Scales utilized in the analysis.

| **Variable Type** | **Variable Name** | **Question(s)** | **Scale** |
| --- | --- | --- | --- |
| Food Security | Food Security- Pre- COVID | Six item USDA Food Security Module- Timeframe "Year before the COVID-19 pandemic" (Before March 2020) | 1= Food Insecure, 0= Food Secure (Answering affirmative to more than 2 of the 6 questions in the module indicates food insecure) |
|  | Food Security- Early COVID | Six item USDA Food Security Module- Timeframe "Since the COVID-19 pandemic" (From March 2020) |  |
|  | Food Security- Later COVID | Six item USDA Food Security Module- Timeframe "In the last four months" (Winter/Spring 2021) |  |
|  | Chronically Food Insecure |  | 1= Food insecure before and since COVID |
|  | Newly Food Insecure |  | 1= Food secure before COVID, food insecure since COVID |
|  | Always Food Secure |  | 1= Food secure before and since COVID |
| Home Food Production Since the COVID-19 pandemic | HFP | Has your household engaged in any of these activities since the COVID-19 outbreak (March 2020)?... | 1= affirmative to any of the 7 specific activities, 0= no affirmatives |
|  | Garden Since | gardening (growing food to eat) | 1= yes, 0= no |
|  | Fishing Since | Fishing or harvesting shellfish to eat |  |
|  | Foraging Since | Foraging (harvesting mushrooms, picking wild or urban fruits or vegetables) |  |
|  | Hunting Since | Hunting or trapping to eat |  |
|  | Livestock Since | Raising animals for meat or dairy |  |
|  | Eggs Since | Keeping poultry for eggs |  |
|  | Preserving Since | Canning, fermenting, drying, smoking or otherwise preserving foods |  |
| More Home Food Production Since the COVID-19 pandemic | HFP More | Pursued any HFP activity for the first time last year or previously did the activity, but did it more last year | 1= Any of the 7 specific activities done for the first time or more last year, 0= activities not changed last year compared to previous years or done less than before |
|  | Garden More | Pursued this activity for the first time last year or previously did the activity, but did it more last year | 1= Done for the first time or more last year, 0= activities not changed last year compared to previous years or done less than before |
|  | Fishing More |  |  |
|  | Foraging More |  |  |
|  | Livestock More |  |  |
|  | Eggs More |  |  |
|  | Preserving More |  |  |
| Demographic Information | Gender identity | Which of the following best describes your gender identity? | 1 = Male, 2 = Female, 3 = Another gender identity, 4 = Prefer not to say |
|  | Race | What is your race? Check all that apply: American Indian/Alaska Native; Asian Indian; Black or African American; Chamorro; Chinese; Filipino; Japanese; Korean; Native Hawaiian; Samoan; Vietnamese; White; |  |
|  | Ethnicity | Are you of Hispanic, Latino, or Spanish origin? | 1 = no, not Hispanic, Latino, or Spanish origin, 2 = Yes, Mexican, Mexican American, Chicano, 3 = Yes, Puerto Rican, 4 = Yes, Cuban, 5 = Yes, Hispanic, Latino, or Spanish origin |
|  | BIPOC |  | 1= BIPOC and/or Hispanic; 0= non-Hispanic White |
|  | Income | Which of the following best describes your household income range in 2019 before taxes? | 1 = Less than $10,000, 2 = $10,000-$24,999, 3 = $25,000-$49,999, 4 = $50,000-$74,999, 5 = $75,000-$99,999, 6 = $100,000 or more |
|  | Job Loss | Have you or anyone in your household experienced a loss of income, reduction of hours, furlough or job loss since the COVID-19 outbreak began March 11th, 2020? | 1= yes, lost job, 0= no job loss |
|  | Education | What is the highest level of formal education that you have completed? | 1 = Some high school (no diploma), 2 = High school graduate (incl. GED), 3 = Some college (no degree), 4 = Associates degree/technical school/apprenticeship, 5 = Bachelor's degree, 6 = Postgraduate (like Master's, PhD) /professional degree (like JD) |
|  | Rural/Urban | What is your zip code? | Zip codes matched to RUCA codes: 1 = urban, 2 = large rural, 3 = small rural, 4 = isolated. Rural categorized as responding 2, 3 or 4 in RUCA code. |

Supplementary Table 2. Results from a matching analysis examining the relationship of HFP activities in the first year of the COVID-19 pandemic to food insecurity during the first year of the COVID-19 pandemic. Reference group is food secure households, therefore a positive coefficient is associated with higher levels of food insecurity.

|  | Coefficient | AI Robust Std. Error | p= | 95% Confidence Interval | | Treated/control raw n= | Treated/control matched n= |
| --- | --- | --- | --- | --- | --- | --- | --- |
| HFP Since COVID | 0.077 | 0.030 | 0.010 | 0.018 | 0.136 | 527/379 | 527/527 |
| Garden Since | 0.059 | 0.030 | 0.051 | -0.003 | 0.118 | 411/472 | 411/411 |
| Fishing Since | 0.090 | 0.047 | 0.058 | -0.003 | 0.183 | 140/743 | 140/140 |
| Foraging Since | 0.172 | 0.049 | 0.000 | 0.076 | 0.267 | 121/762 | 121/121 |
| Hunting Since | 0.014 | 0.047 | 0.003 | 0.048 | 0.234 | 132/751 | 132/132 |
| Livestock Since | 0.189 | 0.062 | 0.003 | 0.065 | 0.312 | 76/807 | 76/76 |
| Eggs Since | 0.192 | 0.051 | 0.000 | 0.093 | 0.292 | 112/771 | 112/112 |
| Preserving Since | 0.127 | 0.039 | 0.001 | 0.051 | 0.204 | 220/663 | 220/220 |
| HFP More | 0.239 | 0.046 | 0.000 | 0.148 | 0.330 | 272/241 | 272/272 |
| Gardens More | 0.176 | 0.048 | 0.000 | 0.082 | 0.270 | 186/224 | 186/186 |
| Fishing More | 0.405 | 0.085 | 0.000 | 0.238 | 0.571 | 52/88 | 52/52 |
| Foraging More | 0.342 | 0.091 | 0.000 | 0.164 | 0.520 | 61/60 | 61/61 |
| Livestock More | 0.247 | 0.123 | 0.045 | 0.006 | 0.488 | 37/38 | 37/37 |
| Eggs More | 0.282 | 0.100 | 0.005 | 0.087 | 0.477 | 62/49 | 62/62 |
| Preserving More | 0.287 | 0.066 | 0.000 | 0.156 | 0.416 | 113/107 | 113/113 |

Supplementary Table 3. Results from a matching analysis examining the relationship of HFP activities in the first year of the COVID-19 pandemic to food insecurity in the last four months before the survey (Winter/Spring 2021). Reference group is food secure households, therefore a positive coefficient indicates that HWFP activity is associated with higher levels of food insecurity if p<0.05.

|  | Coefficient | AI Robust Std. Error | p= | 95% Confidence Interval | | Treated/control raw n= | Treated/control matched n= |
| --- | --- | --- | --- | --- | --- | --- | --- |
| HFP Since COVID | 0.040 | 0.030 | 0.179 | -0.018 | 0.098 | 509/370 | 509/509 |
| Garden Since | 0.023 | 0.030 | 0.441 | -0.036 | 0.082 | 400/461 | 400/400 |
| Fishing Since | 0.110 | 0.047 | 0.018 | 0.018 | 0.201 | 136/725 | 136/136 |
| Foraging Since | 0.175 | 0.049 | 0.000 | 0.079 | 0.271 | 117/744 | 117/117 |
| Hunting Since | 0.136 | 0.047 | 0.004 | 0.043 | 0.228 | 125/736 | 125/125 |
| Livestock Since | 0.203 | 0.065 | 0.002 | 0.075 | 0.331 | 70/791 | 70/70 |
| Eggs Since | 0.189 | 0.052 | 0.000 | 0.087 | 0.291 | 106/755 | 106/106 |
| Preserving Since | 0.110 | 0.038 | 0.004 | 0.034 | 0.186 | 214/647 | 214/214 |
| HFP More | 0.228 | 0.047 | 0.000 | 0.137 | 0.320 | 264/234 | 264/264 |
| Gardens More | 0.185 | 0.045 | 0.000 | 0.095 | 0.275 | 184/216 | 184/184 |
| Fishing More | 0.414 | 0.087 | 0.000 | 0.243 | 0.585 | 50/86 | 50/50 |
| Foraging More | 0.315 | 0.093 | 0.001 | 0.133 | 0.497 | 58/59 | 58/58 |
| Livestock More | 0.203 | 0.126 | 0.107 | -0.043 | 0.450 | 33/37 | 33/33 |
| Eggs More | 0.171 | 0.106 | 0.106 | -0.036 | 0.378 | 59/47 | 59/59 |
| Preserving More | 0.230 | 0.068 | 0.001 | 0.097 | 0.364 | 109/105 | 109/109 |

Supplementary Table 4. Results from a matching analysis examining the relationship of HFP activities in the first year of the COVID-19 pandemic to food insecurity outcomes during the first year of the pandemic. Analyses were conducted within food security groups in the year before the pandemic and compared among those same groups between those doing and not doing HWFP activities during the pandemic. A positive coefficient indicates greater likelihood to be food insecure. For example, the results from row one indicates that among those that were food secure before the pandemic, those that engaged in HWFP activities during the pandemic were positively associated with food insecurity (b=0.054, p=0.042).

| HFP Activity | Food security status Year Before COVID | Coefficient | AI Robust Std. Error | p= | 95% Confidence Interval | | Treated/ control raw n= | Treated/ control matched n= |
| --- | --- | --- | --- | --- | --- | --- | --- | --- |
| HFP Since COVID | Food Secure | 0.054 | 0.027 | 0.042 | 0.002 | 0.107 | 527/379 | 527/527 |
|  | Food Insecure | 0.020 | 0.031 | 0.524 | -0.042 | 0.082 |  |  |
| Garden Since | Food Secure | 0.076 | 0.027 | 0.005 | 0.023 | 0.130 | 411/472 | 411/411 |
|  | Food Insecure | 0.018 | 0.031 | 0.576 | -0.079 | 0.044 |  |  |
| Fishing Since | Food Secure | 0.065 | 0.045 | 0.154 | -0.024 | 0.154 | 140/743 | 140/140 |
|  | Food Insecure | -0.039 | 0.047 | 0.402 | -0.132 | 0.053 |  |  |
| Foraging Since | Food Secure | 0.078 | 0.049 | 0.111 | -0.018 | 0.174 | 121/762 | 121/121 |
|  | Food Insecure | -0.001 | 0.039 | 0.973 | -0.078 | 0.075 |  |  |
| Hunting Since | Food Secure | 0.087 | 0.048 | 0.077 | -0.009 | 0.183 | 132/751 | 132/132 |
|  | Food Insecure | -0.007 | 0.038 | 0.852 | -0.083 | 0.069 |  |  |
| Livestock Since | Food Secure | 0.108 | 0.074 | 0.148 | -0.038 | 0.254 | 76/807 | 76/76 |
|  | Food Insecure | -0.029 | 0.059 | 0.626 | -0.146 | 0.088 |  |  |
| Eggs Since | Food Secure | 0.109 | 0.058 | 0.060 | -0.005 | 0.223 | 112/771 | 112/112 |
|  | Food Insecure | -0.019 | 0.041 | 0.649 | -0.100 | 0.063 |  |  |
| Preserving Since | Food Secure | 0.087 | 0.039 | 0.028 | 0.009 | 0.163 | 220/663 | 220/220 |
|  | Food Insecure | 0.031 | 0.032 | 0.327 | -0.031 | 0.095 |  |  |
|  |  |  |  |  |  |  |  |  |
| HFPMore | Food Secure | 0.162 | 0.043 | 0.000 | 0.077 | 0.246 | 260/239 | 260/260 |
|  | Food Insecure | -0.047 | 0.037 | 0.207 | -0.121 | 0.026 |  |  |
| Garden More | Food Secure | 0.167 | 0.046 | 0.000 | 0.076 | 0.259 | 186/224 | 186/186 |
|  | Food Insecure | 0.021 | 0.057 | 0.710 | -0.091 | 0.133 |  |  |
| Fishing More | Food Secure | 0.315 | 0.112 | 0.005 | 0.096 | 0.535 | 52/88 | 52/52 |
|  | Food Insecure | 0.141 | 0.135 | 0.292 | -0.122 | 0.406 |  |  |
| Foraging More | Food Secure | 0.396 | 0.101 | 0.000 | 0.197 | 0.595 | 61/60 | 61/61 |
|  | Food Insecure | -0.091 | 0.050 | 0.069 | -0.189 | 0.007 |  |  |
| Livestock More | Food Secure | 0.413 | 0.133 | 0.002 | 0.152 | 0.674 | 37/38 | 37/37 |
|  | Food Insecure | -0.037 | 0.125 | 0.763 | -0.282 | 0.207 |  |  |
| Eggs More | Food Secure | 0.305 | 0.101 | 0.003 | 0.107 | 0.503 | 62/49 | 62/62 |
|  | Food Insecure | 0.142 | 0.121 | 0.240 | -0.095 | 0.38 |  |  |
| Preserving More | Food Secure | 0.157 | 0.072 | 0.029 | 0.016 | 0.296 | 113/107 | 113/113 |
|  | Food Insecure | 0.033 | 0.081 | 0.068 | -0.125 | 0.192 |  |  |

Supplementary Table 5. Results from a matching analysis examining the relationship of HFP activities in the first year of the COVID-19 pandemic to food insecurity in the last four months (Late COVID), depending on the food security status of individuals early in the pandemic (Early COVID). Analyses were conducted within food security groups and compared among those same groups between those doing and not doing HWFP activities during the pandemic. A positive coefficient indicates greater likelihood to be food insecure. For example, the results from row one indicates that among those that were food insecure in early COVID, those that engaged in HWFP activities were negatively associated with food insecurity (b=-0.079, p=0.040)- i.e., they were more likely to be food secure in later COVID.

|  | Food Security Status in Early COVID | Coefficient | AI Robust Std. Error | p= | 95% Confidence Interval | | Treated/ control raw n= | Treated/ control matched n= |
| --- | --- | --- | --- | --- | --- | --- | --- | --- |
| HFP Since COVID | Food Secure | -0.001 | 0.012 | 0.967 | -0.025 | 0.024 | 509/370 | 509/509 |
|  | Food Insecure | -0.079 | 0.039 | 0.040 | -0.154 | -0.004 |  |  |
| Garden Since | Food Secure | -0.002 | 0.010 | 0.798 | -0.022 | 0.017 | 400/461 | 400/400 |
|  | Food Insecure | -0.110 | 0.042 | 0.008 | -0.192 | -0.028 |  |  |
| Fishing Since | Food Secure | 0.008 | 0.019 | 0.679 | -0.029 | 0.045 | 136/725 | 136/136 |
|  | Food Insecure | 0.033 | 0.050 | 0.507 | -0.065 | 0.132 |  |  |
| Foraging Since | Food Secure | 0.001 | 0.020 | 0.980 | -0.039 | 0.040 | 117/744 | 117/117 |
|  | Food Insecure | 0.052 | 0.055 | 0.337 | -0.054 | 0.160 |  |  |
| Hunting Since | Food Secure | 0.035 | 0.026 | 0.167 | -0.048 | 0.085 | 125/736 | 125/125 |
|  | Food Insecure | -0.011 | 0.056 | 0.842 | -0.121 | 0.099 |  |  |
| Livestock Since | Food Secure | 0.046 | 0.048 | 0.339 | -0.048 | 0.139 | 70/791 | 70/791 |
|  | Food Insecure | 0.013 | 0.056 | 0.812 | -0.097 | 0.123 |  |  |
| Eggs Since | Food Secure | 0.024 | 0.031 | 0.443 | -0.037 | 0.085 | 106/755 | 106/106 |
|  | Food Insecure | 0.003 | 0.049 | 0.948 | -0.093 | 0.100 |  |  |
| Preserving Since | Food Secure | -0.003 | 0.016 | 0.827 | -0.034 | 0.027 | 214/647 | 214/214 |
|  | Food Insecure | 0.001 | 0.043 | 0.975 | -0.084 | 0.087 |  |  |
|  |  |  |  |  |  |  |  |  |
| HFPMore | Food Secure | 0.008 | 0.012 | 0.501 | -0.015 | 0.031 | 253/232 | 253/253 |
|  | Food Insecure | 0.020 | 0.063 | 0.748 | -0.104 | 0.145 |  |  |
| Gardens More | Food Secure | 0.015 | 0.015 | 0.334 | -0.015 | 0.045 | 184/216 | 184/184 |
|  | Food Insecure | 0.018 | 0.063 | 0.773 | -0.106 | 0.143 |  |  |
| Fishing More | Food Secure | 0.021 | 0.074 | 0.771 | -0.123 | 0.166 | 50/86 | 50/50 |
|  | Food Insecure | -0.023 | 0.068 | 0.738 | -0.156 | 0.111 |  |  |
| Foraging More | Food Secure | 0.063 | 0.061 | 0.302 | -0.056 | 0.181 | 58/59 | 58/58 |
|  | Food Insecure | -0.133 | 0.055 | 0.016 | -0.241 | -0.025 |  |  |
| Livestock More | Food Secure | 0.015 | 0.130 | 0.909 | -0.240 | 0.270 | 33/37 | 33/33 |
|  | Food Insecure | -0.045 | 0.083 | 0.592 | -0.208 | 0.119 |  |  |
| Eggs More | Food Secure | -0.108 | 0.058 | 0.063 | -0.223 | 0.005 | 59/47 | 59/59 |
|  | Food Insecure | -0.041 | 0.077 | 0.601 | -0.193 | 0.111 |  |  |
| Preserving More | Food Secure | 0.002 | 0.029 | 0.948 | -0.055 | 0.059 | 109/105 | 109/105 |
|  | Food Insecure | -0.032 | 0.065 | 0.617 | -0.160 | 0.095 |  |  |

Supplementary Table 6 – Robustness Checks for a matching analysis examining the relationship of HFP activities in the first year of the COVID-19 pandemic to food insecurity during the first year of the COVID-19 pandemic (Original estimates reported in Supplementary Table 2).

|  | Min  Matches | Exact Matches | Coefficient | AI Robust Std. Error | p= | Treated/control raw n= | Treated/control matched n= |
| --- | --- | --- | --- | --- | --- | --- | --- |
| HFP Since COVID | 5 | No | 0.077 | 0.030 | 0.010 | 527/379 | 527/527 |
|  | 4 | No | 0.079 | 0.030 | 0.008 | 527/379 | 527/527 |
|  | 3 | No | 0.071 | 0.030 | 0.019 | 527/379 | 527/527 |
|  | 2 | No | 0.069 | 0.031 | 0.027 | 527/379 | 527/527 |
|  | 1 | No | 0.088 | 0.031 | 0.005 | 527/379 | 527/527 |
|  | 5 | Yes | 0.057 | 0.032 | 0.072 | 448/327 | 448/448 |
|  | 4 | Yes | 0.057 | 0.032 | 0.072 | 448/327 | 448/448 |
|  | 3 | Yes | 0.051 | 0.032 | 0.107 | 464/341 | 464/464 |
|  | 2 | Yes | 0.070 | 0.032 | 0.031 | 485/356 | 485/485 |
|  | 1 | Yes | 0.076 | 0.032 | 0.019 | 512/379 | 512/512 |
|  |  |  |  |  |  |  |  |
| HFP More | 5 | No | 0.239 | 0.046 | 0.000 | 272/241 | 272/272 |
|  | 4 | No | 0.239 | 0.045 | 0.000 | 272/241 | 272/272 |
|  | 3 | No | 0.251 | 0.045 | 0.000 | 272/241 | 272/272 |
|  | 2 | No | 0.265 | 0.046 | 0.000 | 272/241 | 272/272 |
|  | 1 | No | 0.268 | 0.046 | 0.000 | 272/241 | 272/272 |
|  | 5 | Yes | 0.232 | 0.051 | 0.000 | 171/180 | 171/171 |
|  | 4 | Yes | 0.231 | 0.049 | 0.000 | 181/189 | 181/181 |
|  | 3 | Yes | 0.251 | 0.049 | 0.000 | 221/216 | 221/221 |
|  | 2 | Yes | 0.254 | 0.048 | 0.000 | 231/241 | 231/231 |
|  | 1 | Yes | 0.264 | 0.048 | 0.000 | 248/241 | 248/248 |

Supplementary Table 7 – Robustness Checks for matching analysis examining the relationship of HFP activities in the first year of the COVID-19 pandemic to food insecurity in the last four months before the survey (Winter/Spring 2021) (Original estimates reported in Supplementary Table 3).

|  | Min Matches | Exact Matches | Coefficient | AI Robust Std. Error | p= | Treated/control raw n= | Treated/control matched n= |
| --- | --- | --- | --- | --- | --- | --- | --- |
| HFP Since COVID | 5 | No | 0.040 | 0.030 | 0.179 | 509/370 | 509/509 |
|  | 4 | No | 0.048 | 0.030 | 0.107 | 517/372 | 517/517 |
|  | 3 | No | 0.048 | 0.030 | 0.108 | 517/372 | 517/517 |
|  | 2 | No | 0.039 | 0.031 | 0.205 | 517/372 | 517/517 |
|  | 1 | No | 0.058 | 0.031 | 0.061 | 517/372 | 517/517 |
|  | 5 | Yes | 0.034 | 0.032 | 0.280 | 443/321 | 443/443 |
|  | 4 | Yes | 0.034 | 0.032 | 0.280 | 443/321 | 443/443 |
|  | 3 | Yes | 0.033 | 0.031 | 0.287 | 446/326 | 446/446 |
|  | 2 | Yes | 0.042 | 0.032 | 0.193 | 447/372 | 447/447 |
|  | 1 | Yes | 0.048 | 0.032 | 0.139 | 503/372 | 503/503 |
| HFP More | 5 | No | 0.228 | 0.047 | 0.000 | 264/234 | 264/264 |
|  | 4 | No | 0.240 | 0.045 | 0.000 | 270/236 | 270/270 |
|  | 3 | No | 0.253 | 0.044 | 0.000 | 270/236 | 270/270 |
|  | 2 | No | 0.255 | 0.045 | 0.000 | 270/236 | 270/270 |
|  | 1 | No | 0.257 | 0.046 | 0.000 | 270/236 | 270/270 |
|  | 5 | Yes | 0.240 | 0.049 | 0.000 | 171/177 | 171/171 |
|  | 4 | Yes | 0.242 | 0.047 | 0.000 | 185/201 | 185/185 |
|  | 3 | Yes | 0.247 | 0.048 | 0.000 | 222/213 | 222/222 |
|  | 2 | Yes | 0.258 | 0.047 | 0.000 | 231/236 | 231/231 |
|  | 1 | Yes | 0.258 | 0.047 | 0.000 | 246/236 | 246/246 |
|  |  |  |  |  |  |  |  |
|  |  |  |  |  |  |  |  |

Supplementary Table 8 – Robustness checks for matching analysis examining the relationship of HFP activities in the first year of the COVID-19 pandemic to food insecurity in 2020 during the pandemic, depending on the food security status of individuals prior to the pandemic. (Original estimates reported in Supplementary Table 4)

| HFP Activity | Food security status Year Before COVID | Min Matches | Exact | Coefficient | AI Robust Std. Error | p= | Treated/ control raw n= | Treated/ control matched n= |
| --- | --- | --- | --- | --- | --- | --- | --- | --- |
| HFP Since COVID | Food Secure | 5 | No | 0.054 | 0.027 | 0.042 | 376/276 | 376/376 |
|  | Food Insecure | 5 | No | 0.020 | 0.031 | 0.524 | 136/95 | 136/136 |
|  | Food Secure | 1 | No | 0.063 | 0.029 | 0.030 | 376/276 | 376/376 |
|  | Food Insecure | 1 | No | 0.041 | 0.039 | 0.292 | 136/95 | 136/136 |
|  |  |  |  |  |  |  |  |  |
|  | Food Secure | 5 | Yes | 0.044 | 0.027 | 0.106 | 302/225 | 302/302 |
|  | Food Insecure | 5 | Yes | 0.049 | 0.050 | 0.318 | 72/56 | 72/72 |
|  | Food Secure | 1 | Yes | 0.051 | 0.029 | 0.079 | 359/263 | 359/359 |
|  | Food Insecure | 1 | Yes | 0.022 | 0.035 | 0.529 | 116/95 | 116/116 |
|  |  |  |  |  |  |  |  |  |
| HFPMore | Food Secure | 5 | No | 0.161 | 0.043 | 0.000 | 165/202 | 165/165 |
|  | Food Insecure | 5 | No | -0.047 | 0.037 | 0.207 | 95/37 | 95/95 |
|  | Food Secure | 1 | No | 0.176 | 0.045 | 0.000 | 165/202 | 165/165 |
|  | Food Insecure | 1 | No | -0.021 | 0.048 | 0.662 | 95/37 | 95/95 |
|  |  |  |  |  |  |  |  |  |
|  | Food Secure | 5 | Yes | 0.083 | 0.047 | 0.077 | 94/137 | 94/94 |
|  | Food Insecure | 5 | Yes | -0.077 | 0.052 | 0.141 | 26/14 | 26/26 |
|  | Food Secure | 1 | Yes | 0.159 | 0.047 | 0.001 | 147/193 | 193/193 |
|  | Food Insecure | 1 | Yes | -0.033 | 0.065 | 0.609 | 60/37 | 60/60 |
|  |  |  |  |  |  |  |  |  |

Supplementary Table 9 – Robustness checks for matching analysis examining the relationship of HFP activities in the first year of the COVID-19 pandemic to food insecurity in the last four months, depending on the food security status of individuals earlier in the pandemic. (Original estimates reported in Supplementary Table 5)

|  |  |  |  | Coefficient | AI Robust Std. Error | p= | Treated/ control raw n= | Treated/ control matched n= |
| --- | --- | --- | --- | --- | --- | --- | --- | --- |
| HFP Since COVID | Food Secure | 5 | No | -0.0005 | 0.012 | 0.967 | 320/250 | 320/320 |
|  | Food Insecure | 5 | No | -0.079 | 0.039 | 0.040 | 189/115 | 189/189 |
|  | Food Secure | 1 | No | -0.0005 | 0.012 | 0.967 | 320/255 | 320/320 |
|  | Food Insecure | 1 | No | -0.071 | 0.042 | 0.092 | 189/115 | 189/189 |
|  | Food Secure | 5 | Yes | -0.004 | 0.014 | 0.759 | 269/213 | 269/269 |
|  | Food Insecure | 5 | Yes | -0.058 | 0.048 | 0.233 | 98/66 | 98/98 |
|  | Food Secure | 1 | Yes | -0.0005 | 0.012 | 0.964 | 307/246 | 307/246 |
|  | Food Insecure | 1 | Yes | 0.062 | 0.045 | 0.170 | 161/115 | 161/161 |
|  |  |  |  |  |  |  |  |  |
|  |  |  |  |  |  |  |  |  |
| HFPMore | Food Secure | 5 | No | 0.008 | 0.012 | 0.501 | 128/105 | 128/128 |
|  | Food Insecure | 5 | No | 0.020 | 0.063 | 0.748 | 1336/49 | 136/136 |
|  | Food Secure | 1 | No | 0.016 | 0.014 | 0.262 | 128/185 | 128/128 |
|  | Food Insecure | 1 | No | 0.017 | 0.065 | 0.793 | 136/49 | 136/136 |
|  |  |  |  |  |  |  |  |  |
|  | Food Secure | 5 | Yes | 0.002 | 0.015 | 0.888 | 80/124 | 124/124 |
|  | Food Insecure | 5 | Yes | 0.004 | 0.082 | 0.960 | 41/20 | 41/41 |
|  | Food Secure | 1 | Yes | 0.010 | 0.016 | 0.516 | 115/173 | 115/115 |
|  | Food Insecure | 1 | Yes | 0.018 | 0.070 | 0.796 | 92/49 | 92/92 |
|  |  |  |  |  |  |  |  |  |
